# H_2_S Loss through Nalophan™ Bags: Contributions of Adsorption and Diffusion

**DOI:** 10.1155/2017/9690704

**Published:** 2017-06-27

**Authors:** Lidia Eusebio, Laura Capelli, Selena Sironi

**Affiliations:** Department of Chemistry, Materials and Chemical Engineering “Giulio Natta”, Politecnico di Milano, Piazza Leonardo da Vinci 32, 20133 Milano, Italy

## Abstract

Hydrogen-sulfide (H_2_S) is a molecule of small dimensions typically present in the odor emissions from different plants. The European Standard EN 13725:2003 set a maximum storage time allowed of 30 hours, during which the sampling bag has to maintain the mixture of odorants with minimal changes. This study investigates the H_2_S losses through Nalophan bags and it shows that nonnegligible losses of H_2_S can be observed. The percent H_2_S loss after 30 hrs with respect to the initial concentration is equal to 33%  ± 3% at a relative humidity of 20% and equal to 22%  ± 1% at a relative humidity of 60%. The average quantity of adsorbed H_2_S at 30 h is equal to 2.17 10^5^ g_H_2_S_/g_Nalophan_ at a storage humidity of 20% and equal to 1.79 10^5^ g_H_2_S_/g_Nalophan_ at a storage humidity of 60%. The diffusion coefficients of H_2_S through Nalophan, for these two humidity conditions tested, are comparable (i.e., 7.5 10^−12^ m^2^/sec at 20% humidity and 6.6 10^−12^ m^2^/sec at 60% humidity).

## 1. Introduction

Although odors do not have a direct effect on human health, they are considered one of the main causes of discomfort for the population living in areas impacted by odor emissions. Nowadays, olfactory pollution has become a serious environmental concern because it may be the cause of physiological stress to the population [1]. Concerning olfactory nuisance, different European countries have recently adopted specific regulations. The standard methodology for odor concentration measurement is a sensorial technique, that is, dynamic olfactometry [2], which is commonly applied for testing odors for environmental management purposes [3]. This technique is based on the sensation caused by an odorous sample directly on a panel of human assessors [4].

Performing olfactometric analyses on site presents some difficulties. To overcome these problems, the odorous pollutants are collected and stored in appropriate containers until they are analyzed in an olfactometric laboratory [4–6]. In order to regulate the quality of the olfactometric analysis, the European Standard on dynamic olfactometry [2] defines the requirements for the materials used for sampling equipment. The requirements determined by the EN13725 for the olfactometry materials are as follows: being odorless and being able to minimize the physical or chemical interaction between sample components and sampling materials and having low permeability in order to minimize sample losses caused by diffusion and smooth surface.

The materials allowed by EN13725 for sample containers (i.e., bags) are as follows: tetrafluoroethylene hexafluoropropylene copolymer (FEP); Tedlar™ (polyvinyl fluoride, PVF), and Nalophan (polyethylene terephthalate, PET). Moreover, European Standard set a maximum storage time allowed, during which the sampling bag has to maintain the mixture of odorants with minimal changes.

Since the publication of the Standard in 2003, several studies have been carried out in order to test the characteristics of the materials listed in the EN 13725 [2] and to verify their suitability for olfactometric measurements. In Table 1 literature studies are reported investigating losses of odorous molecules through sampling bags [1, 5–36].

More in detail, in Table 1, beside the author and year, the polymer film studied, the thickness of the film, the pollutant taken into account and the detection system adopted are reported.

The results of the studies reported in Table 1 underline that the chemical pollutants diffused through the polymeric film are mainly small molecules, like ammonia (NH_3_) and H_2_S.

Nalophan is generally the most used material for the manufacturing of sampling bags for olfactometric analyses, due to its inert properties and cost-effectiveness. Despite these advantages, it is known in literature that Nalophan allows the diffusion of specific compounds, such as water [15]. Water can diffuse quickly through the Nalophan polymeric film because of its structure [15]. The results of the studies reported in Table 1 showed that the chemical compounds that diffuse through the Nalophan film are water, NH_3_, and H_2_S [1, 9, 10, 15, 27]; the last two compounds diffuse easily because these molecules have dimensions similar to water [1, 9, 10, 15, 27].

H_2_S and NH_3_ are typically odorous pollutants present in the emissions from several plants such as solid waste and waste water treatment.

In this paper, the attention was focused on H_2_S, a malodorous compound with smell similar to rotten eggs. H_2_S is detected by human olfaction at very low concentrations—about 1 ppb [37–39]—and it is typically found in the emissions from different plants, like industry [30], agriculture [16, 31], waste water treatment [7], and waste treatment [21].

Generally, the articles present in literature (Table 1) focus the attention mainly on the H_2_S loss by determining the H_2_S recovery in the sampling bag.

The study of the contribution of pollutant losses, such as diffusion and adsorption, is not easy because the diffusion through the polymeric film is influenced by the nature of the polymer as well as by the nature of the diffusing pollutant [1, 40].

More in detail, the polymer characteristics that influence the diffusion processes are as follows: the chemical nature of the polymer, its crystalline structure and orientation, the free volume, the molecular cohesion, the relative humidity, temperature, hydrogen bonding, polarity, solubility parameter, and solvent size and shape [40].

As reported by Klopffer and Flaconneche in 2001 [41], the polymer structure plays an important role in the determination of the transport phenomena through the polymeric film.

It is well known in literature that transport phenomena of small molecules through an amorphous polymer are governed by mechanisms of adsorption and diffusion [40]. Transport phenomena can be decomposed into five successive stages (Figure 3) [40, 41]: (i) the diffusion through the boundary layer of the side corresponding to the higher partial pressure (upstream side); (ii) the adsorption of the gas (by chemical affinity or by solubility) on the polymer; (iii) the diffusion of the gas inside the polymer's membrane; (iv) the desorption of the gas at the side of lower partial pressure; and (v) the diffusion through the limit layer of the downstream side.

Only few studies in literature [1, 14, 27, 28] have faced the problem of diffusion through the sampling bags by calculating the diffusion coefficient of the inspected chemical compound. Moreover, in most studies, the amount of chemical compound lost due to adsorption on the polymeric film has been neglected. Adsorption can be neglected when high concentrations are considered (e.g., 50000 ppm NH_3_ by Sironi et al. (2014) [1, 27, 28]), whereas for medium-low concentrations (e.g., in the range of ppb to few ppm) the effect of adsorption becomes significant. In this study, both the effects of diffusion through the polyethylene terephthalate (PET, Nalophan) film and the adsorption on the film are investigated. The experiments described in this paper aim to investigate the relative contributions of the two phenomena causing H_2_S loss in Nalophan bags, that is, adsorption and diffusion. The evaluations were carried out by calculating the amount of H_2_S adsorbed in the Nalophan film and the diffusion coefficient *D* relevant to this material. Finally, the influence of physical parameter like relative humidity (RH) on both the diffusion coefficient and the adsorption was evaluated.

## 2. Materials and Methods

### 2.1. Materials

**Figure 1:**
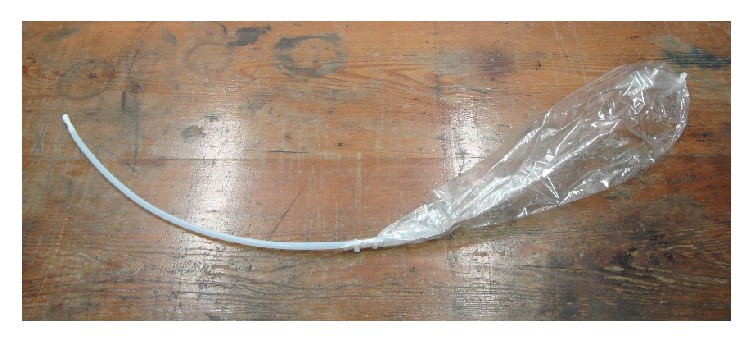
Nalophan sampling bag, capacity 6 liters.

The sampling bags studied with capacity of 6 liters are prepared from a tubular film of Nalophan supplied by Tilmmanns S.p.A. and shown in Figure 1. The polymer film consists of a 20-*µ*m thick one-layer foil.

The H_2_S decay over time was evaluated by measuring the H_2_S concentration inside the bag over time by means of a high performance miniature sensor able to detect H_2_S at ppb level. More in detail, the sensor used for the H_2_S concentration measurement is a CairClip apparatus, developed by Cairpol, a French start-up (Alès Engineer School of Mines), which consists in amperometric detection with a dynamic air sampling system, a special filter, and a high sensitive electronic circuit containing a data logger [42]. The instrument was calibrated by the manufacturer and it has a life-cycle of one year. The accuracy of this instrument declared by the manufacturer is 10 ppb, in a range between 30 and 1000 ppb of H_2_S and mercaptans.

All the test samples were prepared by filling the Nalophan bags with a gaseous mixture of 800 ppb_V_ of H_2_S in air, defined as the “test mixture” in the paper. The samples were obtained by withdrawing the H_2_S from a certified H_2_S gas cylinder (SAPIO technical gas, Milano, Italy) into Nalophan bags with a volume of 6 liters and a surface of 2580 cm^2^.

**Figure 2:**
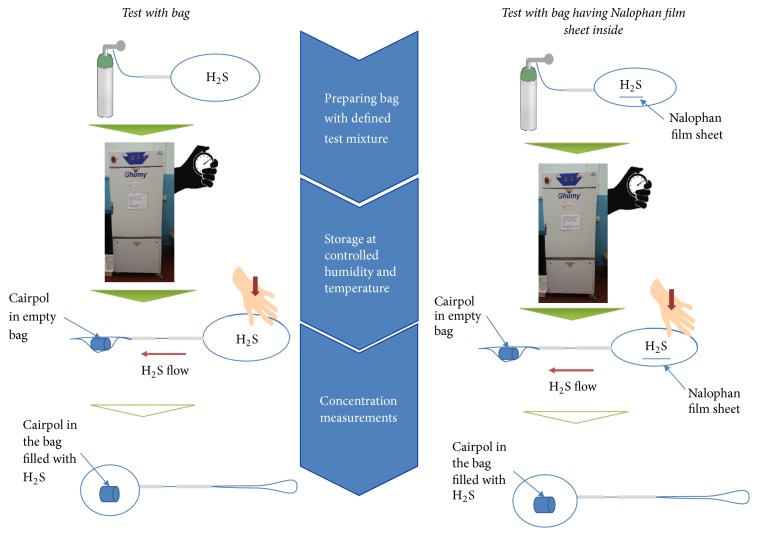
Scheme of the method adopted.

One aspect that had to be considered for the design of the experiment is that the CairClip has steel parts that may interact with the H_2_S and reduce its concentration, thereby affecting the measurements of the H_2_S concentration decay through the Nalophan, which is the aim of this paper. Therefore, in order to avoid undesired interactions of the CairClip sensor with the H_2_S during the sample storage period, the concentration measurements were carried out by moving the gaseous mixture contained in the storage bag into another identical empty bag containing the CairClip sensor (Figure 2). Because of the short time of the measurement, the adsorption/diffusion effect in this bag is assumed to be negligible. In order to evaluate the H_2_S concentration decay over time, this procedure had to be repeated for different time intervals. A new bag had to be prepared for each tested interval and then its contents transferred to the bag containing the measurement apparatus after the desired time interval (Figure 2).

The H_2_S concentration after each tested time interval was then compared to the initial H_2_S concentration in the test mixture (800 ppb) in order to evaluate the H_2_S loss over time.

During storage, external physical parameters like temperature (i.e., 23°C) and relative humidity (i.e., RH% equal to 20 and 60, resp.) were kept under control using a climatic chamber (Chamber GHUMY by Fratelli Galli, Milano, Italy).

### 2.2. Methods

In order to evaluate the contribution of adsorption and the diffusion phenomena into the Nalophan bags, several tests had to be performed, and three replications of each condition and time were tested, following the scheme in Figure 2.

After a first test using a bag with a volume of 6 liters and a surface of 2580 cm^2^ (in the following defined as “B-no film”), other tests were repeated using bags with the same geometrical characteristics (i.e., volume of 6 liters and a surface of 2580 cm^2^), in which sheet of film of the same material (i.e., a 20 *μ*m thick Nalophan sheet) was inserted. Three different tests were performed by changing the dimensions of the sheet of film inserted inside the bag. This way, besides the “B-no film” with no film in it, three different types of bags were prepared:

- Nalophan bag with volume of 6 L and surface of 2580 cm^2^ containing a sheet of film of 1900 cm^2^ (in the following defined as “B-film 1900”).
- Nalophan bag with volume of 6 L and surface of 2580 cm^2^ containing a sheet of film of 2580 cm^2^ (in the following defined as “B-film 2580”).
- Nalophan bag with volume of 6 L and surface of 2580 cm^2^ containing a sheet of film of 3520 cm^2^ (in the following defined as “B-film 3520”).

The idea of inserting the sheets of Nalophan of different dimensions inside identical bags had the aim of evaluating the contribution of adsorption of the H_2_S in the Nalophan film, which is expected to increase with the surface of the Nalophan film the H_2_S is in contact with.

Table 2 reports the experimental conditions tested.

The tests were conducted by measuring the H_2_S concentration at different storage time intervals, as explained in the previous paragraph. The time intervals tested were from 0 to 30 hrs, the latter being the maximum storage time allowed by the reference standard EN 13725:2003. All measurements, reported in Table 2, were repeated three times each.

The test temperature of the samples was fixed at 23°C. The role of humidity on the H_2_S concentration decay inside the bag was evaluated by storing the bags at different external humidity values, of 20% and 60%, respectively.

A suitable procedure had to be adopted in order to normalize the Nalophan films tests in terms of initial conditions of water absorbed. In fact, Nalophan is proven to be water permeable [15], and thus the water adsorption in the film is connected to the external environmental conditions. For this reason, in order to normalize the water content of the tested Nalophan films, all bags were stored for 12 hours at the test conditions using a climatic chamber before the beginning of the tests.

This procedure allows obtaining repeatable results by reducing the measurement errors related to the state of swelling of the polymer matrix.

The comparison of the H_2_S residual concentration inside the bag after the tested storage time with the initial H_2_S concentration in the test mixture allowed the evaluation of the H_2_S loss over time. As already mentioned, the aim of this paper was not only the quantification of the H_2_S loss over time but also the evaluation of the relative contribution of adsorption and diffusion to this loss. H_2_S adsorption was evaluated using (12) to (14) (see § Calculations), whereas diffusion was calculated based on Fick's law. To calculate the diffusion coefficient *D* of H_2_S through Nalophan, (15) to (17) were used (see § Calculations). The measurements were performed at different times and the diffusion coefficient *D* was averaged over 30 hours.

### 2.3. Calculations

The model used to determine the H_2_S loss, due to both adsorption and diffusion, starts from the method developed in Sironi et al. 2014 [1] by adapting this for H_2_S. More in detail, the novelty of this work is to separate the two contributions on pollutants loss from the sampling bag: adsorption on polymeric matrix and diffusion through the film.

The diffusion phenomenon through a polymeric film can be described by Fick's law. Accordingly the specific molar flow is defined as

(1) $$\begin{array}{c} j=-D\frac{\partial C}{\partial x}, \end{array}$$

where

- *j* is the specific molar flow (mol/m^2^/sec),
- *D* is the diffusion coefficient of the compound through the film (m^2^/sec),
- *C* is the concentration of the diffusing compound (mol/m^3^),
- *x* is the differential thickness of the polymeric film of the bag.

The thickness of polymeric film of the bag can therefore be expressed as

(2) $$\begin{array}{c} \overset{z}{\underset{0}{\int }}dx=z, \end{array}$$

where *z* is the thickness (m) of the polymeric film of the bag.

**Figure 3:**
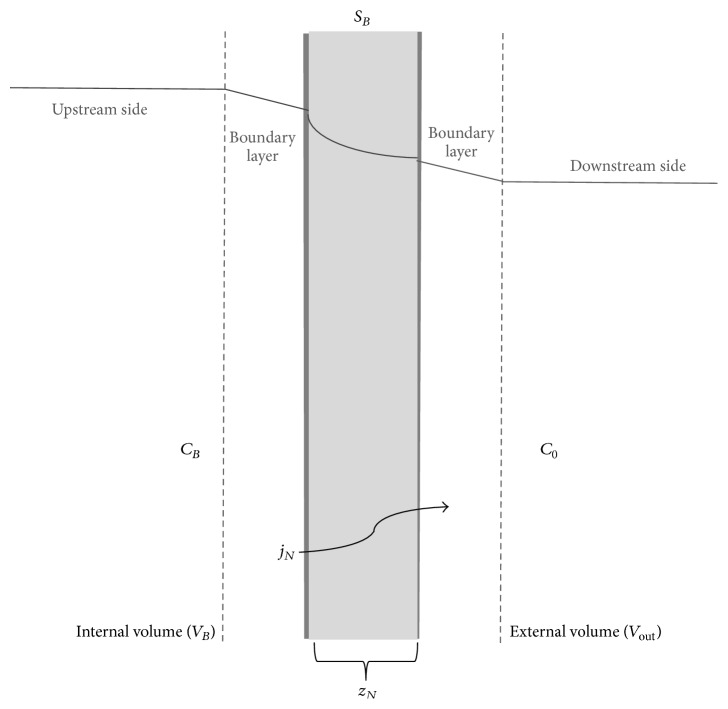
Schematization of diffusion through the thin film of the bag.

Referring to Figure 3, which schematizes the diffusion phenomenon through the thin film that constitutes the sampling bag, it is possible to define the following:

- *S*_*B*_ is the surface of the polymeric film of the bag (m^2^).
- *Z*_*B*_ is the thickness of the polymeric film of the bag (m).
- *C*_*B*_ is the concentration in the inside volume (mol/m^3^).
- *C*_0_ is the concentration outside the film (mol/m^3^), and for a single bag it is generally considered negligible (*C*_0_ = 0).
- *j* is the specific molar flow through the polymeric film of the bag (mol/m^2^/sec), assuming in first approximation *j* constant along the film (*x*).

By integrating (1) in *dx* between 0 and *z*_*B*_, the specific molar flow *j* can be expressed as

(3) $$\begin{array}{c} j=-D\frac{C_{0}-C_{B}}{z_{B}}, \end{array}$$

where *j* is relevant to an infinitesimal portion of the exchange surface *dS*.

Assuming that the internal molar concentration *C*_*B*_ is homogeneous inside the whole internal volume *V*_*B*_ and also the external concentration *C*_0_ is constant inside the external volume, then the global flow *J* through the exchange surface *S*_*B*_ can be calculated by integrating as follows:

(4) J=∫0SBj dS,

(5) J=SBj.

Combining (3) with (5), the molar flow through the surface can be expressed as

(6) $$\begin{array}{c} \frac{\partial M_{B}}{\partial t}=-\frac{\partial C_{B}V_{B}}{\partial t}=-\frac{S_{B}D}{z_{B}}\left(\;C_{B}-C_{0}\;\right). \end{array}$$

If the external concentration *C*_0_ is assumed to be equal to zero (*C*_0_ = 0), which is the case if the bag is placed in a neutral environment (where the presence of H_2_S may be considered negligible), (6) can be rewritten as

(7) $$\begin{array}{c} -\frac{\partial C_{B}V_{B}}{\partial t}=-\frac{S_{B}D}{z_{B}}C_{B}. \end{array}$$

According to this model, the concentration decay over time turns out to be a function of the surface area (*S*_*B*_), the volume of the sampled gas *V*_*B*_, the film thickness (*z*_*B*_), the time (*t*), the diffusion coefficient (*D*) that depends on the characteristics of the material, and the concentration gradient through the polymeric barrier (Δ*C*).

The boundary conditions considered for the integration of (7) are

(8) CB=Cfor  t=t∗,CB=Cinfor  t=0.

The integration of (7) allows computing the concentration trend over time:

(9) ln⁡CCin=−SBDVBzBt,CCin=e−SBD/VBzBt.

The H_2_S loss (percent) through the bag over time can be expressed as

(10) $$\begin{array}{c} H_{2}S_{loss\%}=\left(\;1-\frac{C_{t_{i}}}{C_{in}}\;\right)*100, \end{array}$$

where *C*_*t*_*i*__ is the concentration measured at time *t*_*i*_ and *C*_in_ is the initial concentration.

The loss of H_2_S is due both to adsorption in the Nalophan and to diffusion through the bag walls.

The H_2_S loss due to these phenomena can be calculated as the difference between the initial amount of H_2_S (H_2_S_in_) and the amount measured at the time *t*_*i*_ (H_2_S_*t*_*i*__):

(11) $$\begin{array}{c} {H_{2}S}_{loss}\left(\;\mu g\;\right)={H_{2}S}_{in}\left(\;\mu g\;\right)-{H_{2}S}_{t_{i}}\left(\;\mu g\;\right). \end{array}$$

In order to evaluate the relative contributions of the two phenomena (adsorption and diffusion) to the H_2_S loss inside the Nalophan bag, the following system has to be solved:

(12) H2Sloss_1μg=a∗SB+y,H2Sloss_2μg=a∗SB+a∗Sfilm+y,

where

- H_2_S_loss_1_ is the amount of H_2_S loss at time *t*_*i*_ (*µ*g) measured for the simple Nalophan bag,
- H_2_S_loss_2_ is the amount of H_2_S loss at time *t*_*i*_ (*µ*g) measured for the Nalophan bag with the Nalophan sheets inserted,
- *a* is the contribution of the adsorbed H_2_S (*µ*g/m^2^),
- *y* is the contribution of the diffused H_2_S (*µ*g),
- *S*_*B*_ is surface area of the bag (m^2^),
- *S*_film_ is surface area of the sheet of film inserted in the bag (m^2^).

The first equation of the system refers to the test condition in which the bag has no additional film inserted in it. On the contrary, the second equation refers to the bags containing the sheets of Nalophan film. Moreover, it is important to notice that using the same thickness of the film (i.e., 20 *µ*m) the data are expressed in terms of surface unit. Therefore, the data obtained are directly correlated to the data expressed in terms of mass unit.

The adsorbed amount per unit of surface (H_2_S_adsorbed_/m^2^) can be obtained by subtracting the contribution of the diffusion (i.e., *y*) from the amount of H_2_S losses at time *t*_*i*_ (i.e., H_2_S_loss_ (*µ*g)), according to (12):

(13) $$\begin{array}{c} \frac{{H_{2}S}_{adsorbed}}{m^{2}}=\frac{{H_{2}S}_{loss}\left(\mu g\right)-y}{S_{B}+S_{film}}. \end{array}$$

The adsorbed amount (H_2_S_adsorbed_) related to the considered surface can be obtained by multiplying H_2_S_adsorbed_/m^2^ by the inner film surface (i.e., *S*_film_):

(14) $$\begin{array}{c} {H_{2}S}_{adsorbed}=\frac{{H_{2}S}_{adsorbed}}{m^{2}}S_{film}. \end{array}$$

The diffused amount (i.e., H_2_S_diff_) was calculated as the difference between the H_2_S amount losses (H_2_S_loss_) at time *t*_*i*_ and the adsorbed amount:

(15) $$\begin{array}{c} {H_{2}S}_{diff}={H_{2}S}_{loss}-{H_{2}S}_{adsorbed}. \end{array}$$

The diffusion coefficient *D*_*t*_*i*__ for each time interval *t*_*i*_ was calculated according to the following equation:

(16) $$\begin{array}{c} D_{t_{i}}=-\frac{V_{B}z_{B}}{S_{B}t_{i}}\ln\left(\;\frac{{H_{2}S}_{diff}}{{H_{2}S}_{in}}\;\right), \end{array}$$

where *t*_*i*_ is the time interval and H_2_S_diff_ is the concentration diffused at time *t*_*i*_.

The diffusion coefficient of H_2_S through Nalophan was finally calculated as the average of the different values of *D*_*t*_*i*__ weighted on the corresponding storage time *t*_*i*_:

(17) $$\begin{array}{c} \overset{-}{D}=\frac{\sum_{i}D_{t_{i}}t_{i}}{\sum_{i}t_{i}}. \end{array}$$

## 3. Results and Discussion

As previously mentioned, the main objective of this study was the estimation of the relative contribution of the two phenomena (i.e., adsorption and diffusion) that are responsible for the H_2_S concentration decay inside Nalophan bags used for olfactometric sampling.

Table 3 shows the ratio *C*_*t*_*i*__/*C*_in_, where *C*_*t*_*i*__ is the H_2_S concentration measured at different time intervals (*t*_*i*_) normalized to the initial concentration (*C*_in_), and the percent loss of H_2_S (%) with respect to the initial concentration. The storage temperature was fixed at 23°C and the relative humidity was 20% and 60%, respectively. Table 3 reports the results obtained for the simple Nalophan bag (“B-no film”) and the other three bags prepared by inserting sheets of Nalophan of different dimensions inside the bags, that is, 1900 cm^2^ (“B-film 1900”), 2580 cm^2^ (“B-film 2580”), and 3520 cm^2^ (“B-film 3520”), respectively, as described in the Methods.

The percent loss of H_2_S (%) (Table 3) inside the bag with respect to the initial concentration over time was calculated according to (10). The H_2_S concentration decay is due to both the adsorption into the Nalophan (i.e., both the bag itself and the inserted film sheet) and the diffusion through the bag walls.

The percent loss of H_2_S (%) from the simple bag that does not contain the extra Nalophan film sheet in it (“B-no film”) after 30 hr turns out to be equal to about 33%  ± 3% at a storage humidity of 20% and equal to 22%  ± 1% at a storage humidity of 60%. This trend is coherent with other data reported in the scientific literature dealing with the same subject. As an example, a study by Akdeniz et al. (2011) [7], also dealing with H_2_S losses through polymeric films (Tedlar and Flex Foil), reports losses of about 20% after 36 hours.

Moreover, it is possible to observe for the single bag how the data show that the trends of the H_2_S losses (%) are little bit higher decreasing the storage relative humidity. This is due to the presence of water caused by the humidity gradient, as already observed in Sironi et al. (2014a, b) [1, 27].

The data reported in Table 3 show also an increase of the H_2_S losses (%) increasing the surface of the polymeric film sheet inserted in the bag. The H_2_S percent loss (%), at a storage humidity of 20%, after 30 hr turns out to be equal to 47% for the bag containing the film sheet with a surface of 1900 cm^2^, increasing up to 71% for the bag containing the film sheet with a surface of 3520 cm^2^. The same trend is observed at a storage humidity of 60%: the H_2_S percent loss (%) after 30 hr turns out to be equal to 46% for the bag containing the film sheet with a surface of 1900 cm^2^, increasing up to 63% for the bag containing the film sheet with a surface of 3520 cm^2^.

**Figure 4:**
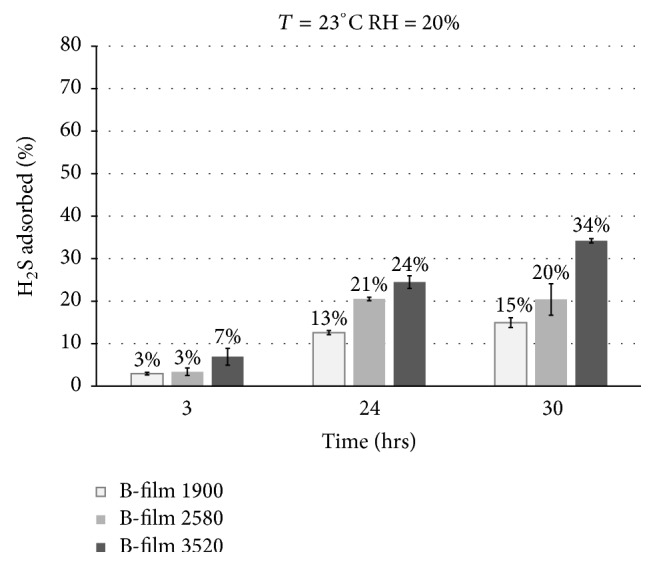
Adsorbed H_2_S (%) at specific time intervals at a storage temperature of 23°C and humidity of 20%. The bag tested was with the film sheets inside. The surface of the internal film sheet was equal to 1900 cm^2^ (B-film 1900), 2580 cm^2^ (B-film 2580), and 3520 cm^2^ (B-film 3520), respectively. The data reported are the average of the results from three different tests performed at the same conditions.

**Figure 5:**
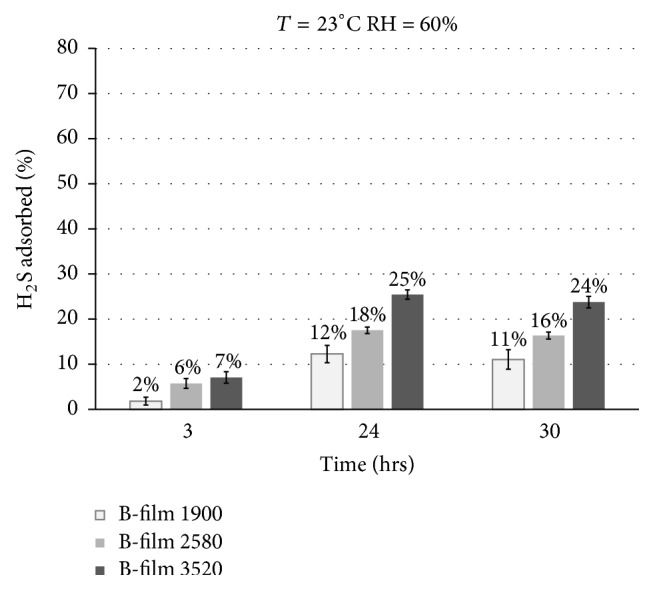
Adsorbed H_2_S (%) at specific time intervals at storage temperature of 23°C and humidity of 60%. The bag tested was with the film sheets inside. The surface of the internal film sheet was equal to 1900 cm^2^ (B-film 1900), 2580 cm^2^ (B-film 2580), and 3520 cm^2^ (B-film 3520), respectively. The data reported are the average of the results from three different tests performed at the same conditions.

As said above, the H_2_S losses (%) inside the bag with respect to the initial concentration are affected by two contributions: adsorption into the Nalophan and diffusion through the Nalophan bag walls. In order to evaluate these two contributions separately, the H_2_S ratio adsorbed into the Nalophan film was evaluated as the ratio between H_2_S_adsorbed_ (estimated according to (14)) and the initial concentration (H_2_S_in_). Figures 4 and 5 report the adsorbed H_2_S (%) at specific time intervals at a storage temperature of 23°C and a humidity of 20% and 60%, respectively.

As it is possible to observe in Figure 4 and in Figure 5, the ratio of adsorbed H_2_S (%) increases by increasing the inner film sheet surface. The adsorbed H_2_S (%) at a storage humidity of 20% (Figure 4) after 30 hr turns out to be equal to

- about 15% for the bag containing the film sheet with a surface of 1900 cm^2^ (“B-film 1900”),
- about 20% for the bag containing the film sheet with a surface of 2580 cm^2^ (“B-film 2580”),
- about 34% for the bag containing the film sheet with a surface of 3520 cm^2^ (“B-film 3520”).

The adsorbed H_2_S (%) at a storage humidity of 60% (Figure 5) after 30 hr turns out to be equal to

- about 11% for the bag containing the film sheet with a surface of 1900 cm^2^ (“B-film 1900”),
- about 16% for the bag containing the film sheet with a surface of 2580 cm^2^ (“B-film 2580”),
- about 24% for the bag containing the film sheet with a surface of 3520 cm^2^ (“B-film 3520”).

The data reported above show a weak increase of the ratio of adsorbed H_2_S (%) for the bag stored at low humidity (i.e., 20%). The Nalophan film is made with PET (polyethylene terephthalate) that is known from literature to be water permeable [15]. Therefore, when storing the bag at high humidity (i.e., 60%), the amount of water that can be adsorbed on the film is greater compared to the storage condition at low humidity (i.e., 20%). At a temperature of 23°C and relative humidity of 20% the partial pressure of water is equal to 4 mmHg, whereas at a temperature of 23°C and relative humidity of 60% the partial pressure of water is equal to 13 mmHg. Therefore, in this second condition, it is likely that the water is adsorbed on the polymer matrix instead of the H_2_S (competitive adsorption).

**Figure 6:**
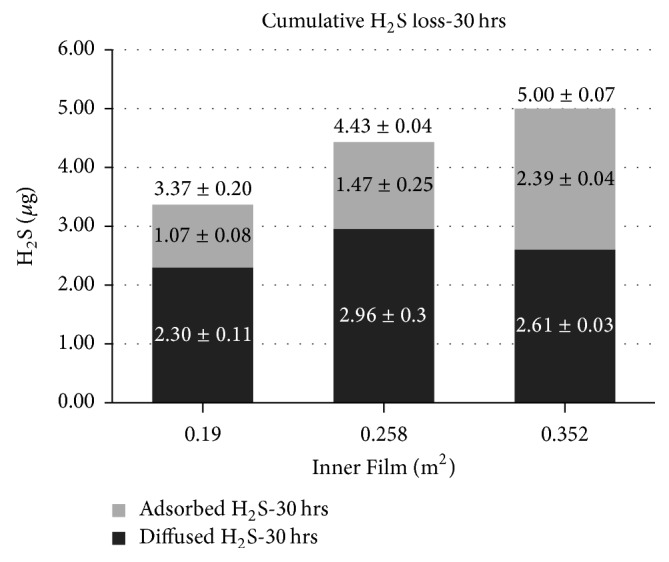
The amount of H_2_S in terms of cumulative losses, diffusion losses, and adsorption losses related to the surface of the inner film at a storage temperature of 23°C and humidity of 20%. The data reported are the average of the results from three different tests performed at the same conditions.

**Figure 7:**
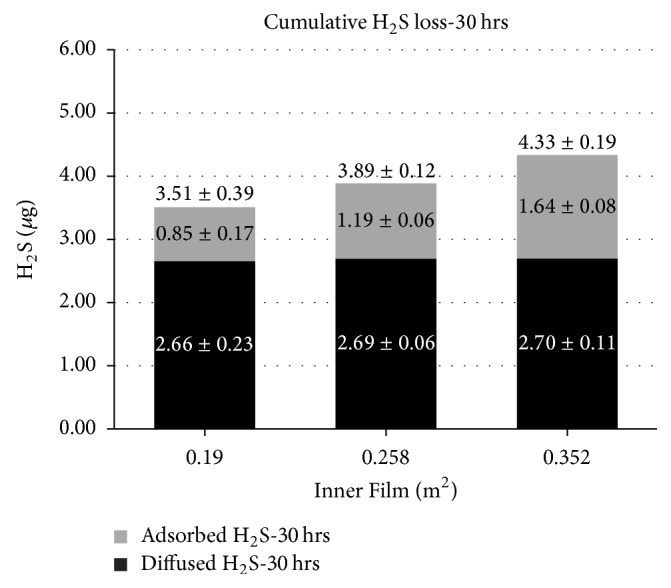
The amount of H_2_S in terms of cumulative losses, diffusion losses, and adsorption losses related to the surface of the inner film at a storage temperature of 23°C and humidity of 60%. The data reported are the average of the results from three different tests.

Figures 6 and 7 illustrate the amount of H_2_S in terms of cumulative losses (*µ*g) and the two contributions, that is, on one hand the H_2_S adsorbed on the polymeric film and on the other hand the H_2_S diffused trough the bag walls. The results are shown in function of the surface area of the Nalophan film sheet inserted inside the test bags at a storage humidity of 20% and 60%, respectively.

As expected, the amount of H_2_S that is adsorbed increases by increasing the surface of the Nalophan film sheet inserted inside the bag. Also, the contribution of diffusion remains almost constant for the two values of relative humidity tested (i.e., RH 20% and 60%, resp.). This aspect was expected because the film sheet inserted has no internal concentration gradient (Δ*C*) (see Fick law (7)).

Moreover, it is possible to observe that diffusion is predominant compared to adsorption, although the latter is not negligible. The only exceptions are observed at a temperature of 23°C and a relative humidity of 20% in the bag containing the Nalophan film sheet with a surface of 3520 cm^2^ (“B-film 3520”) (Figure 6), since in these conditions the contribution of diffusion is comparable to that of adsorption.

The averaged data of the adsorbed amount per surface unit (H_2_S_adsorbed_/m^2^) in *µ*g/m^2^ (see (12)) at specific times (i.e., 3 hr, 24 hr, and 30 hr) are reported in Table 4.

It is possible to observe (Table 4) that the results at 24 hours and 30 hours relevant to both the storage conditions tested present comparable values of H_2_S_adsorbed_/m^2^. At 3 hr, the value of H_2_S_adsorbed_/m^2^ is lower. The averaged values relevant to 24 and 30 hr of H_2_S_adsorbed_/m^2^ are equal to 5.8 *µ*g/m^2^ (which corresponds to a ratio H_2_S_adsorbed (g)_/g_Nalophan_ equal to 2.17 10^5^ g_H_2_S_/g_Nalophan_) at a relative humidity of 20% and to 4.8 *µ*g/m^2^ at a relative humidity of 60% (which corresponds to a ratio H_2_S_adsorbed (g)_/g_Nalophan_ equal to 1.79 10^5^ g_H_2_S_/g_Nalophan_), respectively. The value of H_2_S_adsorbed_/g was obtained by combining the value of H_2_S_adsorbed_/m^2^ with the thickness of the film, which is equal to 20 *µ*m, and the density of amorphous PET, which is equal to 1.335 g/cm^3^ [43].

As already observed, at a storage humidity of 20% the amount of adsorbed H_2_S is higher than the adsorbed amount at the storage humidity of 60%. This may be due to the fact that to a relative humidity of 60% corresponds a higher amount of water, given that the water can compete with the H_2_S in the adsorption on the polymeric film. Therefore, it is possible to assert that the adsorption of H_2_S on the polymeric film is influenced by the storage humidity.

Moreover, the data in Table 4 show that after three hours of storage the polymeric film is not yet saturated. The steady state conditions, at which the polymer film is completely saturated, are reached at 24 hours. The steady state is considered reached when the sorption amount of H_2_S does not vary with time in analogies with Fick law [41]. Therefore, in order to calculate the diffusion coefficient (*D*) only the data acquired at 24 hours and 30 hours were used. The diffusion coefficient was evaluated according to (16).

Table 5 reports the diffusion coefficient *D*_*t*_*i*__ for each time interval *t*_*i*_ at a storage temperature of 23°C and a humidity of 20% and 60%, respectively.

The diffusion coefficient of H_2_S ($\overset{-}{D}$) through Nalophan is finally calculated as the average of the different values of *D*_*t*_*i*__ (Table 5) weighted on the corresponding storage time *t*_*i*_ according to (17).

The resulting value for $\overset{-}{D}$, at a storage humidity of 20%, is equal to 7.5 10^−12^ m^2^/sec with a standard deviation equal to 1.2 10^−14^ m^2^/sec.

The resulting value for $\overset{-}{D}$, at a storage humidity of 60%, is equal to 6.6 10^−12^ m^2^/sec with a standard deviation equal to 7.9 10^−15^ m^2^/sec.

The resulting values for $\overset{-}{D}$ obtained at two different storage conditions (i.e., humidity of 20% and of 60%., resp.) present the same order of magnitude.

## 4. Conclusions

The H_2_S losses from the Nalophan bag always turned out to be significant. The H_2_S loss after 30 hr was equal to 33% at a relative humidity of 20% and equal to 22% at a relative humidity of 60%.

The average value of H_2_S_adsorbed_/m^2^ turns out to be equal to 5.8 *µ*g/m^2^ at a storage humidity of 20% and equal to 4.8 *µ*g/m^2^ at a storage humidity of 60%.

The contribution of the adsorption phenomenon, under the test conditions evaluated, is less significant than the diffusion, though not negligible. When increasing the surface of the film sheet inserted in the bag (i.e., test with “B-film 3520” at a humidity of 20%) then the contribution of adsorption to the H_2_S loss inside the bag becomes comparable with the contribution of diffusion. Therefore, in the case of medium-low concentrations as it happens for those tests (from few ppb to few ppm), an increase of the polymeric surface produces an increase in the H_2_S loss due to the adsorption on the polymeric film. As a consequence, in order to reduce the adsorption phenomena on the polymeric film when storing gases like H_2_S at medium-low concentrations (i.e., in a range of ppb to few ppm), it is better to reduce the contact surface exposed to the gas using small sampling bags and storing the bag at a high relative humidity (i.e., RH% equal to 60%). During sampling of H_2_S, in order to reduce the odor losses, special care should be taken when the expected H_2_S concentration is medium or low (e.g., in the range of ppb to few ppm) because the adsorption phenomena on the polymer film in this case are not negligible.

The diffusion coefficients of H_2_S through Nalophan, for these two humidity conditions tested, are comparable (i.e., 7.5 10^−12^ m^2^/sec at 20% humidity and 6.6 10^−12^ m^2^/sec at 60% humidity).

Evaluating the two contributions of H_2_S loss (i.e., adsorption and diffusion) is important to choose the best sampling strategy (i.e., the choice of the bag material), as well as the most proper storage time and conditions.

In order to reduce the diffusion phenomena through the bag, it is possible to use polyethylene terephthalate (i.e., commercial named Nalophan) coupled with foils. Nevertheless, this choice does not solve the problems linked to the loss by adsorption of H_2_S on the polymeric matrix.

## Figures and Tables

**Table 1:** Scheme of the studies related to the pollutant loss through sampling bag.

Reference number	Author and year	Polymeric Film	Thickness [*µ*m]	Chemical compound	Detection System
[1]	Sironi et al., 2014	Nalophan	20	NH_3_	GC

[5]	Y.-H. Kim and K.-H. Kim, 2012	PEA	n.d.	Benzene, toluene, styrene, p-xylene, methyl ethyl ketone, methyl isobutyl ketone, isobutyl alcohol, butyl acetate, acetaldehyde, propionaldehyde, butyraldehyde, isovaleraldehyde, valeraldehyde	GC MS

[6]	Laor et al., 2010	Tedlar	n.d.	Odors emitted from municipal sewage, aeration basin, sludge, livestock manure, coffee	DO
		Nalophan	20		

[7]	Akdeniz et al., 2011	Tedlar	n.d.	NH_3_, CH_4_, N_2_O, H_2_S, total sulfur dioxide	pulsed fluorescence analyzer, chemiluminescence analyzer, GC IR
		FlexFoil	n.d.		

[8]	Bakhtari, 2014	Nalophan	50	Benzene, ozone, H_2_S	DO
		Tedlar	50		
		Teflon	50		

[9]	Beghi and Guillot, 2006	Tedlar	50	Methanol, ethanol, acetone, n-propanol, n-hexane, dichloroethane, trichloroethane, methyl isobutyl ketone, toluene, butyl acetate	GC
		Teflon	50		
		FlexFoil	75		

[10]	Beghi and Guillot, 2008	Nalophan	20	Acetone, n-propanol, ethanol, n-hexane, 1,2-dichloroethane, trichloroethane, methyl isobutyl ketone, toluene, butyl acetate, ethylbenzene	GC
		Tedlar	50		

[11]	Boeker et al., 2014	Nalophan	n.d.	Butylamine, ethylamine, carbon disulfide, dimethyl sulfide, butyl acetate, ethyl acetate, n-butyrate acetate, dichloroethane, chloroform, dichloromethane, 2-heptanone, methyl isobutyl ketone, ethyl methyl ketone, acetone, n-hexyl acetate, *α*-ionone, limonene, *α*-pinene, 1,2,3,4-tetraidronaphtene, ethylbenzene, toluene, skatole, indole, methanol, p-cresol, phenol, n-hexanol, n-butanol, ethanol, *α*-hexyl cinnamaldehyde, furfural, hexanal	GC MS
		NaloSafe	n.d.		
		Nalobar	n.d.		
		Tedlar	n.d.		

[12]	Bokowa, 2012	Tedlar	n.d.	2-Methylbutane, pentane, 2,2-dimethylbutane, 2-methylpentane, cyclopentane, 3-methylpentane, 1-hexene, hexane, 2,4-dimethylpentane, methylcyclopentane, 3-methyl-1-hexene, 3-methyl-1, 3-pentadiene, 2-methylhexane, 2,3-dimethyl pentane, cyclohexane, 3-methylhexane, benzene, cyclohexene, heptane, 2,5-dimethylhexane, methyl cyclohexane, ethyl cyclopentane, 2-methylpentane, 3-methylpentane, 2,3,5-trimetilesano, t-1,4-dimethylcyclohexane, toluene, octane, 1,1-dimethylcyclohexane, t-1,2-dimethylhexane, c-1,4-dimethylcyclohexane, propyl cyclopentane, c-1, 2-dimethylcyclohexane, 2 + 4-methyloctane, 3-methyloctane, ethylbenzene, nonane, m + p-xylene, 3,7-dimethyl-1-octene, o-xylene, cumene, propylbenzene, dean, 1,3,5-trimethylbenzene, 1,2,4-trimethyl benzene, p-cymene, 1,2,3-trimethyl benzene, undecane, dodecane, tridecane, tetradecane	GC MS

[13]	Cariou and Guillot, 2006	Tedlar	50	2-Propanolo, 2-butanone, toluene	GC

[14]	Eusebio et al., 2016	Nalophan	20 *μ*m	H_2_S	specific H_2_S sensors

[15]	Guillot and Beghi, 2008	Nalophan	20	H_2_S, H_2_O	GC
		Tedlar,	50		
		Teflon,	50		
		FlexFoil	75		

[16]	Hansen et al., 2011	Tedlar,	50	Carboxylic acids, phenols, indoles, sulfur compounds	GC MS
		Nalophan	20		

[17]	Jo et al., 2012	PEA	n.d.	H_2_S, methanethiol, carbon disulfide, SO_2_, dimethyl sulfide, dimethyl disulfide	GC
		Tedlar	n.d.		

[18]	Kim, 2006	Tedlar	n.d.	H_2_S, methanethiol, dimethyl sulfide, dimethyl disulfide	GC
		Polyester	n.d.		

[19]	Kim et al., 2012	PEA,	50	Benzene, toluene, p-xylene, styrene, methyl ethyl ketone, methyl isobutyl ketone, butyl acetate, isobutyl alcohol	GC MS
		Tedlar	50		

[20]	Koziel et al., 2005	Tedlar,	50	Acetic acid, propionic acid, isobutyric acid, butyric acid, isovaleric acid, valeric acid, hexanoic acid, p-cresol, indole, 4-ethylphenol, 2-aminoacetophenone	GC MS
		Teflon,	50		
		foil	125		
		Melinex (PET)	15		

[21]	Le et al., 2013	Tedlar	n.d.	H_2_S, methanethiol, ethanethiol, dimethyl sulfide, tert-butanethiol, ethyl methyl sulfide, 1-butanethiol, dimethyl disulfide, diethyl disulfide, dimethyl trisulfide	GC
		Mylar	n.d.		
		Nalophan	n.d.		

[22]	Le et al. 2015	Tedlar	50	Hydrogen sulfide, methanethiol, ethanethiol, dimethyl sulfide, tert-butanethiol, ethyl methyl sulfide, 1-butanethiol, dimethyl disulfide, diethyl disulfide, dimethyl trisulfide	GC
		Mylar	25		
		Nalophan	25		

[23]	Mochalski et al., 2009	Nalophan,	20	H_2_S, methanethiol, ethanethiol, carbonyl sulfide, dimethyl sulfide, carbon disulfide	GC MS
		Tedlar transparent,	50		
		Tedlar black,	25		
		Teflon,	n.d.		
		FlexFoil	n.d.		

[24]	Mochalski et al., 2013	Tedlar	50	n-Butane, n-pentane, n-hexane, n-octane, n-decane, isobutane, 3-methyl pentane, 2-butene E and Z, 2-pentene E and Z, 1-hexene, methylcyclopentane, a-pinene, (+)-3-carene, p-cymene, D-limonene, eucalyptol, benzene, toluene, p-xylene, o-xylene, acetone, 2-butanone, 2-pentanone, 4-heptanone, 2-butenone, propanal, 2-methyl propanal, butanal, hexanal, octanal, 2-methyl-2-propenal, furan, 2-methyl furan, 2,5-dimethyl furan, thiophene, 3-methyl thiophene, methyl acetate, ethyl acetate, n-propyl acetate, methyl methacrylate, dimethyl selenide, ethyl ether, pyrimidine, acetonitrile, 2-methyl pentane, 4-methyl heptane, isoprene, ethylbenzene, dimethyl sulfide, 2-methyl-1-pentene, n-butyl acetate, 2,4-dimethyl heptane, 2,4-dimethyl-1-heptene, 4-methyl octane, 3-methyl furan, methyl propyl sulfide	GC MS
		Kynar	50.8		
		Flexfilm	76		

[25]	Parker et al., 2010	Tedlar	n.d.	p-Cresol, acetic acid, propionic acid, isobutyric acid, butyric acid, isovaleric acid, valeric acid, hexanoic acid	DO/GC-MS

[26]	Sáiz et al., 2011	Polyethylene	n.d.	Dynamites	GC MS HPLC

[27]	Sironi et al., 2014	Nalophan	20	NH_3_	GC

[28]	Sironi et al., 2014	Nalophan	20	NH_3_	GC

[29]	Sulyok et al., 2002	Silcosteel cylinder	n.d.	Methylmercaptan, ethylmercaptan, Dimethyl sulfide, 2-Propylmercaptan, 1-Propylmercaptan, 2-Butylmercaptan, 1-Butylmercaptan	GC
		Tedlar	n.d.		

[30]	Sulyok et al., 2001	Silcosteel cylinder	n.d.	Methylmercaptan, ethylmercaptan, dimethyl sulfide, ethyl methyl sulfide, 2-propylmercaptan, 1-propylmercaptan, 2-butylmercaptan, diethyl sulfide, 1-butylmercaptan	GC
		Tedlar	50		
		Tedlar black/clear layered	50		

[31]	Trabue et al., 2006	Tedlar	n.d.	Agricultural odorants, acetic acid, propanoic acid, 2-methylpropanoicacid, butanoic acid, 3-methylbutanoic acid, pentanoic acid, 4-methylpentanoic acid, hexanoic acid, heptanoic acid, phenol, 4-methylphenol, 4-ethylphenol, indole, 3-methylindole, Volatile fatty acid, phenol, 4-methylphenol, 4-ethylphenol, indole, and 3-methylindole	GC MS

[32]	Van Harreveld, 2003	Nalophan	20	Tobacco	DO
		Cali-5-Bond coated Nalophan	131		

[33]	Van Durme and Werbrouck, 2015	Nalophan	20 *μ*m	Japanese Indoor Air Standard mix	GC MS

[34]	Wang et al., 2011	Nalophan	40	H_2_O (gas)	QCM sensors
		Nalophan-CF_4_	125		

[35]	Zarra et al., 2012	Nalophan	25	WWTP odorants	DO
		Tedlar	50		
		Teflon	50		

[36]	Zhu et al., 2015	Tedlar	n.d.	Ethylmercaptan, butyric acid, isovaleric acid, p-cresol	GC MS
		Metallized-FEP	n.d.		

GC gas chromatography, MS mass spectrometry, PEA Polyester aluminium, WWTP waste water treatment plant, DO dynamic olfactometry, HPLC liquid chromatography, QCM quartz-crystal-microbalance.

**Table 2:** Experimental conditions. The bag tested was without any film inside (B-no film) and with the film inside. The surface of the internal film sheet was equal to 1900 cm^2^ (B-film 1900), 2580 cm^2^ (B-film 2580), and 3520 cm^2^ (B-film 3520) respectively.

Test code	Bag capacity [L]	Bag surface [cm^2^]	Film sheet surface [cm^2^]
B-no film	6	2580	No film inside
B-film 1900	6	2580	1900
B-film 2580	6	2580	2580
B-film 3520	6	2580	3520

**Table 3:** Experimental data relevant to the H_2_S loss over time in a Nalophan bag stored at temperature of 23°C and humidity of 20% and 60%. The bag tested was without any film inside (B-no film) and with the film inside. The surface of the internal film sheet was equal to 1900 cm^2^ (B-film 1900), 2580 cm^2^ (B-film 2580), and 3520 cm^2^ (B-film 3520), respectively. The data reported are the average of the results from three different tests performed at the same conditions.

	Time [hr]	*T*23°C RH% 20		*T*23°C RH% 60	
		*C*_*t*_*i*__/*C*_in_	% H_2_S losses	*C*_*t*_*i*__/*C*_in_	% H_2_S losses
B-no film	3	0.92 ± 0.04	8% ± 4%	0.96 ± 0.02	4% ± 2%
	24	0.77 ± 0.02	23% ± 2%	0.80 ± 0.004	20% ± 0.4%
	30	0.67 ± 0.03	33% ± 3%	0.78 ± 0.011	22% ± 1.1

B-film 1900	3	0.89 ± 0.01	11% ± 1%	0.94 ± 0.02	6% ± 2%
	24	0.65 ± 0.01	35% ± 1%	0.60 ± 0.045	40% ± 4.5%
	30	0.53 ± 0.03	47% ± 3%	0.54 ± 0.051	46% ± 5.1%

B-film 2580	3	0.89 ± 0.02	11% ± 2%	0.87 ± 0.02	13% ± 2%
	24	0.54 ± 0.01	46% ± 1%	0.53 ± 0.015	47% ± 1.5%
	30	0.39 ± 0.002	61% ± 0.2%	0.47 ± 0.016	53% ± 1.6%

B-film 3520	3	0.84 ± 0.04	16% ± 4%	0.86 ± 0.02	14% ± 2%
	24	0.53 ± 0.03	47% ± 3%	0.44 ± 0.017	56% ± 1.7%
	30	0.28 ± 0.01	71% ± 1%	0.37 ± 0.020	63% ± 2%

**Table 4:** Averaged data of the amount of H_2_S adsorbed per surface unit (H_2_S_adsorbed_/m^2^). The bag tested was without any film inside (B-no film) and with the film inside. The surface of the internal film sheet was equal to 1900 cm^2^ (B- film 1900), 2580 cm^2^ (B- film 2580), and 3520 cm^2^ (B- film 3520), respectively. The data reported are the average of the results from three different tests performed at the same conditions.

		RH% 20			RH% 60		
		3 hrs	24 hrs	30 hrs	3 hrs	24 hrs	30 hrs
H_2_S_adsorbed_/m^2^ [*µ*g/m^2^]	B-film 1900	1.11 ± 0.12	4.73 ± 0.19	5.65 ± 0.45	0.74 ± 0.35	4.98 ± 0.78	4.48 ± 0.88
	B-film 2580	0.95 ± 0.26	5.75 ± 0.07	6.94 ± 0.08	1.62 ± 0.29	4.95 ± 0.15	4.62 ± 0.23
	B-film 3520	1.38 ± 0.39	4.87 ± 0.3	6.80 ± 0.12	1.38 ± 0.27	4.97 ± 0.27	4.65 ± 0.30

**Table 5:** Diffusion coefficient of H_2_S over time in a Nalophan bag stored at a temperature of 23°C and a humidity of 20% and 60%, respectively. The bag tested was without any film inside (B-no film) and with the film inside. The surface of the internal film sheet was equal to 1900 cm^2^ (B-film 1900), 2580 cm^2^ (B-film 2580), and 3520 cm^2^ (B-film 3520), respectively.

	Time [hr]	*T*23°C RH% 20		*T*23°C RH% 60	
		*C*_diff_/*C*_0_	*D*_*t*_*i*__ (m^2^/sec)	*C*_diff_/*C*_0_	*D*_*t*_*i*__ (m^2^/sec)
B-no film	24	5%	1.61*E* − 11	12%	1.15*E* − 11
	24	5%	1.62*E* − 11	12%	1.16*E* − 11
	24	5%	1.60*E* − 11	12%	1.16*E* − 11
	30	12%	9.06*E* − 12	12%	9.21*E* − 12
	30	12%	9.14*E* − 12	12%	9.27*E* − 12
	30	12%	8.96*E* − 12	12%	9.29*E* − 12

B-film 1900	24	22%	8.07*E* − 12	25%	7.42*E* − 12
	24	22%	8.05*E* − 12	30%	6.41*E* − 12
	24	21%	8.35*E* − 12	27%	6.96*E* − 12
	30	31%	5.02*E* − 12	33%	4.82*E* − 12
	30	31%	5.00*E* − 12	38%	4.18*E* − 12
	30	34%	4.65*E* − 12	33%	4.78*E* − 12

B-film 2580	24	26%	7.29*E* − 12	30%	6.47*E* − 12
	24	25%	7.39*E* − 12	29%	6.66*E* − 12
	24	25%	7.48*E* − 12	28%	6.76*E* − 12
	30	37%	4.33*E* − 12	37%	4.29*E* − 12
	30	43%	3.63*E* − 12	38%	4.19*E* − 12
	30	43%	3.63*E* − 12	36%	4.38*E* − 12

B-film 3520	24	24%	7.64*E* − 12	30%	6.42*E* − 12
	24	22%	8.10*E* − 12	32%	6.20*E* − 12
	24	22%	8.05*E* − 12	31%	6.29*E* − 12
	30	38%	4.21*E* − 12	39%	4.10*E* − 12
	30	37%	4.28*E* − 12	40%	3.94*E* − 12
	30	37%	4.28*E* − 12	39%	4.04*E* − 12
